# Maternal Knowledge of Oral Health During Pregnancy and Early Childhood: A Systematic Review

**DOI:** 10.4317/jced.63650

**Published:** 2026-01-28

**Authors:** Alicia Aznar-Marín, María Dolores Casaña-Ruiz, Alfredo Perales-Marín, Montserrat Catalá-Pizarro

**Affiliations:** 1 Department of Stomatology, Faculty of Medicine and Dentistry, University of Valencia, 46010 Valencia, Spain; 2 Department of Obstetrics and Gynecology, La Fe University and Polytechnic Hospital, 46026 Valencia, Spain. Department of Pediatrics, Obstetrics and Gynecology, Faculty of Medicine and Dentistry, University of Valencia, 12006 Valencia, Spain

## Abstract

**Background:**

Pregnant women's knowledge about the changes that occur in the oral cavity during pregnancy, as well as their impact on the course of pregnancy, is insufficient. Likewise, there is limited awareness of early childhood caries and of preventive oral-health measures.

**Objectives:**

The aim of this study is to assess the level of knowledge, attitudes, and practices related to oral health among pregnant women, and to identify the main knowledge gaps concerning their own oral health and that of their future children.

**Data sources:**

Scopus, Web of Science, Embase, and PubMed electronic databases were searched.

**Eligibility criteria:**

Observational and cross-sectional studies published in different languages were included. Studies assessing adult pregnant women's knowledge, attitudes, and practices regarding their own oral health and that of their children were selected. Studies were excluded if they were qualitative in nature or focused solely on beliefs and opinions; included fewer than 50 participants; addressed topics unrelated to the review objectives; or were conducted after an informational intervention. Studies focusing exclusively on children, or on women who were neither primiparous nor recently delivered, were also excluded. In addition, studies were excluded if the questionnaire or its individual items were not reported, or if a questionnaire was used without a quantitative assessment of knowledge.

**Material and Methods:**

Given the heterogeneity of the questionnaires and scoring systems, a quantitative synthesis was not feasible; therefore, a narrative synthesis approach was adopted. The methodological quality of the included studies was assessed using the Newcastle-Ottawa Quality Assessment Scale (NOS) for longitudinal studies, and modified Newcastle-Ottawa Scale (NOS) for cross-sectional studies.

**Results:**

A total of 26 studies met the inclusion criteria. Studies reporting scoring systems showed knowledge levels ranging from 0.13-8.39 (out of a maximum of 12 points) or 40-82.8% correct answers, depending on the assessment format. Overall, most pregnant women demonstrated insufficient knowledge regarding pregnancy-related oral changes, periodontal disease, and their potential adverse effects on gestation, as well as the risk of transmitting cariogenic bacteria to the newborn. Persistent misconceptions were identified including the belief that "a tooth is lost with every pregnancy," and incorrect perceptions regarding the safety of dental treatments during pregnancy. Furthermore, a substantial proportion of women were unaware of when to initiate infant oral hygiene or schedule their child´s first dental visit. Oral hygiene practices were often irregular, and information was obtained from dentists, gynecologists, magazines, and the internet; however, access to structured professional counseling remains limited.

**Limitations:**

The review was restricted to studies published in English, French, Italian, and Spanish, which may have limited representation from non Western or low resource settings. Grey literature and non indexed sources were not included, potentially leading to exclusion of locally relevant evidence. Considerable variability in study populations and methodologies affected comparability across studies. In addition, many investigations relied on non validated questionnaires (42%), which likely contributed to the observed heterogeneity in quality scores (40-82.8%).

**Conclusions:**

These findings highlight the need to strengthen oral health education during pregnancy through integrated strategies and the active involvement of dentists within prenatal care programs.

## Introduction

Pregnancy, defined as the period of fetal gestation within the maternal uterus (1), involves a series of physiological and hormonal changes that significantly modify a woman's body, including the oral cavity. Understanding these transformations is essential for anticipating their impact on the mother-child dyad, identifying potential associated problems, and determining the most appropriate timing for their management. Maintaining good oral health before and during pregnancy helps protect women's general health and quality of life, while also reducing the transmission of pathogenic bacteria from mother to child (2). The American Academy of Pediatric Dentistry (AAPD) acknowledges that perinatal and early childhood oral health constitutes the foundation upon which preventive education and dental care should be built, with the aim of increasing the likelihood that children grow up free from preventable oral diseases. In this process, the family plays a crucial role as the primary learning environment where children acquire knowledge, attitudes, and habits related to oral health (3). Despite the relevance of oral health during pregnancy, many women do not seek dental care during this stage. Nevertheless, this period represents a critical window of opportunity to promote oral care habits in both expectant mothers and their infants. In some contexts, it may also be the only time when women can access dental services. Barriers to improving oral health and dental service utilisation among pregnant women and their children are multifactorial, involving both healthcare system-related factors and individual determinants (4). In recent years, the international community has increasingly recognized the role of prenatal care providers-such as family physicians, midwives, gynecologists, and obstetricians-in implementing preventive oral health strategies within their clinical practice, as reflected in clinical guidelines and practice protocols (5). This systematic review aims to assess pregnant women's oral health knowledge, identify key knowledge gaps, and examine variables associated with knowledge levels, based on the available scientific evidence.

## Material and Methods

The present study was conducted following the guidelines of the PRISMA (Preferred Reporting Items for Systematic Reviews) 2020 statement (6). It was registered in PROSPERO under the following reference: [CRD42023406157]. 1. Study Question and Eligibility Criteria The PICO question was: "What is the level of knowledge about oral health during pregnancy among pregnant women?" Eligibility criteria were established according to the PICO model as follows: Population (P): Pregnant women. Intervention (I): No informational intervention. Comparison/Control (C): Not applicable. Outcome (O): Knowledge, attitudes, and practices. 2. Inclusion and Exclusion Criteria Cross-sectional observational studies published in English, French, Italian, or Spanish that assessed baseline knowledge, attitudes, and oral health practices during pregnancy among adult pregnant women were included. Qualitative studies or those focused exclusively on beliefs and opinions were excluded, as well as studies that included a limited number of knowledge-related questions (3 items) or samples of fewer than 50 participants, and those addressing unrelated topics or conducted after an informational intervention. Studies focused solely on children or on women who were not primiparous or not newly delivered were also excluded, as were studies that did not provide the questionnaire or the items used for the assessment in the publication, and those that, despite using a questionnaire, did not evaluate knowledge qualitatively. 3. Data Sources and Search Strategy In January 2023, an electronic search was conducted in the Scopus, Web of Science (WOS), Embase, and PubMed databases. The search terms used were: (mother OR wom OR mater*) AND (pregnan*) AND (knowledge OR awareness) AND ("oral health") *, combined using the Boolean operators "AND" and "OR", and applied to the title, abstract, and keyword fields. The selection of terms was based on previous studies in this field. In addition, after the selection of the articles, a manual review of the references of the included studies was carried out to identify any further relevant research. The search was last updated on 17 April 2025. The advanced search equations and the results obtained for the individual and combined fields are presented in Table 1.

Table 1

4. Study Screening and Selection Process After searching each database, the records were imported into the EndNote Reference Manager (version 21.2), and duplicates were removed. Subsequently, two independent reviewers (A-AM and A-PM) performed the initial screening of the titles and abstracts. When the abstract did not provide sufficient information to determine whether the study should be included or excluded, the full text was assessed. Discrepancies between reviewers (which accounted for approximately 10% of the studies during the screening phase) were resolved by consensus. When consensus could not be reached, a third reviewer (M-CP) was consulted. In the second phase, the full texts of the selected articles were evaluated to determine their final eligibility according to the established inclusion and exclusion criteria. 5. Data Extraction and Recorded Variables A table was prepared containing the variables to be recorded for each study. Two independent reviewers (A-AM and MD-CR) performed the data extraction in parallel. Any discrepancies were resolved by consensus or, if necessary, through consultation with a third reviewer (M-CP). The extracted data included Study characteristics: author, year of publication, country or study location. Sociodemographic characteristics: age, parity, education, employment status. Questionnaire characteristics: type (self-developed non-validated; validated/reliability-tested), structure (closed-ended multiple-choice; subscales for knowledge), and topics covered (oral hygiene during pregnancy, mother-to-child bacterial transmission, early childhood caries prevention, dental visits, nutrition, fluoride use). Knowledge outcomes: overall mean scores and specific domains (pregnancy-related oral changes/periodontal disease/adverse gestation effects, cariogenic bacteria transmission to newborns, infant oral hygiene timing/first dental visit, dental treatment safety during pregnancy, misconceptions e.g., "a tooth lost per pregnancy"). The results of this extraction are presented in Table 2.

Table 2

6. Quality Assessment To assess the methodological quality of the included studies, the Newcastle-Ottawa Quality Assessment Scale (NOS) (33) was used for longitudinal studies, and the modified Newcastle-Ottawa Scale (NOS) (34 , 35) was applied for cross-sectional studies. This tool is structured into three domains: selection (sample representativeness, non-response rate, and instrument validation), comparability, and outcomes (assessment and statistical analysis). For longitudinal studies, each domain can achieve a specific maximum score: 4 points for selection, 2 points for comparability, and 3 points for outcomes, with a maximum total score of 9 points per study. For cross-sectional studies, the maximum possible score for each domain is: 5 points for selection, 2 points for comparability, and 3 points for outcomes, resulting in a maximum total score of 10 points per study. The results of the assessment are presented in Table 3 and Table 4.

Table 3

Table 4

7. Data Synthesis Due to heterogeneity in the questionnaires and scoring systems employed, a quantitative synthesis (meta-analysis) was not feasible; therefore, a narrative synthesis was undertaken. No statistical pooling was performed, and heterogeneity was not formally assessed (e.g., using the I² statistic). Given the narrative approach and the clinical and methodological heterogeneity of the included cross-sectional studies, no formal assessment of publication bias (e.g., funnel plots or Egger's test) or certainty of evidence (e.g., GRADE) was conducted.

## Results

**Figure 1:**
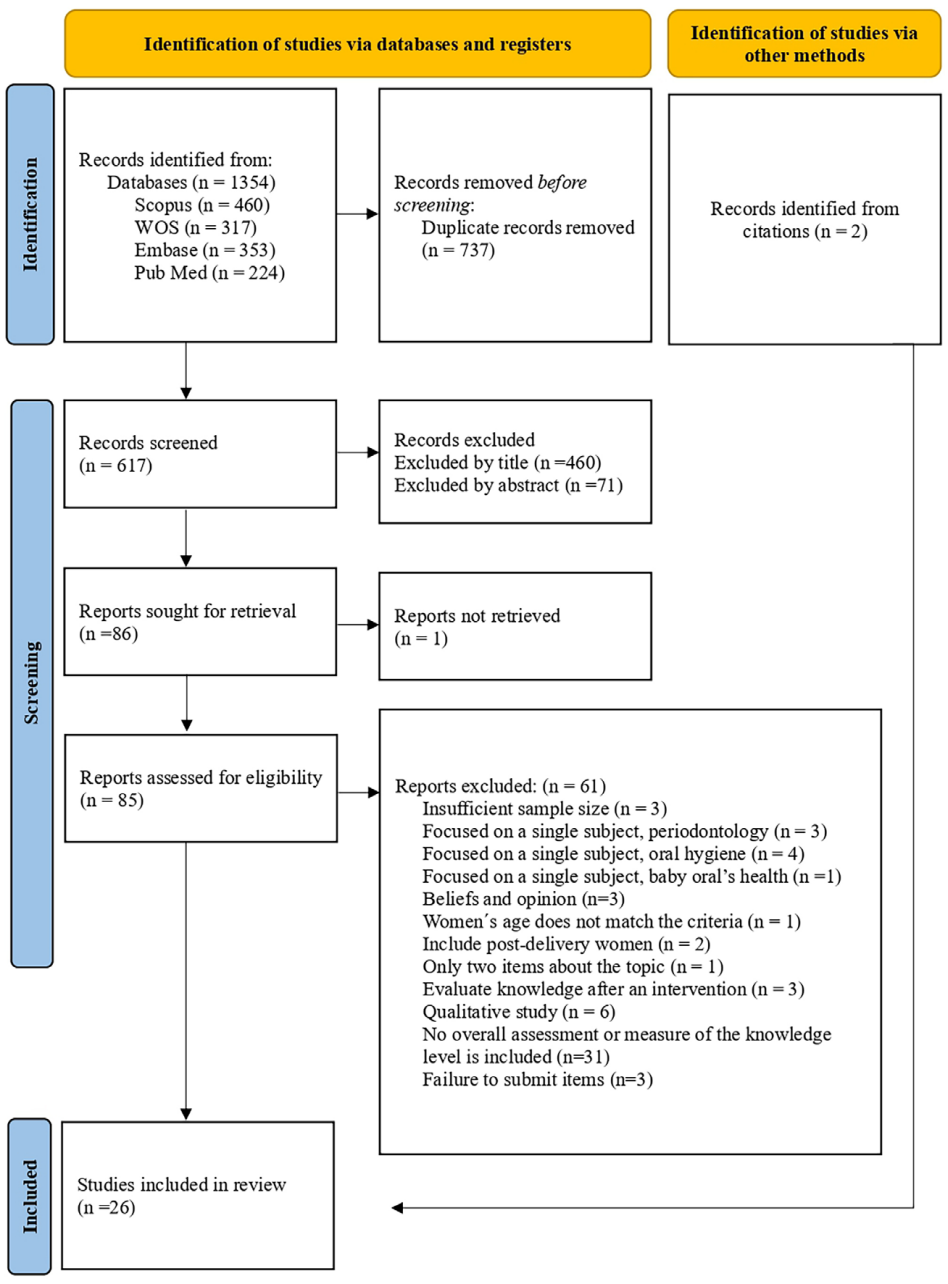
PRISMA flow diagram.

1. Search Results and Study Selection A total of 1.354 studies were identified through the electronic databases: Scopus (n = 460), Web of Science (n = 317), Embase (n = 353), and PubMed (n = 224). After removing 737 duplicate records, 617 studies remained for evaluation. During the initial screening, 460 records were excluded after reading the titles and 71 after reviewing the abstracts, leaving 86 potentially eligible studies. One of these could not be retrieved in full text. A total of 85 articles were assessed in full, of which 61 were excluded for not meeting the inclusion criteria. This process resulted in the selection of 24 studies. Additionally, two studies were identified through reference list screening, yielding a final total of 26 studies included in the review. Figure 1 presents the PRISMA flow diagram, detailing the stages of identification, screening, selection, and inclusion of the studies.

1

2. Results of the Studies Given the substantial heterogeneity in study populations, questionnaire designs, scoring systems, and outcome definitions, the results are synthesized using a qualitative and descriptive narrative approach. No pooled estimates, summary measures, or graphical representations (e.g., forest plots) are presented, as quantitative comparison across studies was not feasible. 2.1. Variables related to oral health knowledge It should be noted that the classification of knowledge levels (e.g., low, moderate, high) was defined by the original authors of each study and was based on study-specific cut-off points. Given the differences in questionnaire length, scoring ranges, and validation status, these qualitative categories are not directly comparable across studies and should be interpreted within the context of each individual investigation. The included studies primarily assessed the level of maternal knowledge about oral health during pregnancy and/or early childhood. This variable was measured using structured questionnaires, most of which were self-developed and non-validated (n = 11) (7 - 12 , 19 , 21 - 23 , 28), although 15 studies (13 - 18 , 20 , 24 - 27 , 29 - 32) employed validated instruments or reported reliability analyses (e.g., Pearson correlation coefficient or prior pilot testing). Two studies did not report this information. Overall findings revealed wide variability in maternal knowledge levels, influenced by sociodemographic factors such as education, age, and previous maternal experience. Studies that used validated instruments and larger samples (15 , 17 , 29) demonstrated greater methodological rigor and higher mean scores, suggesting a trend toward improved knowledge in settings with active preventive programs. Sociodemographic variables. The most frequently described variables were as follows: Maternal age ranged between 26 and 32 years on average, with minimum values of 24 years (19 , 24 , 30) and maximums of 35 years (15 , 29). Educational level was commonly categorized as primary, secondary, technical/university, or postgraduate, with most studies reporting a positive association between higher education and better oral health knowledge. Parity was classified as nulliparous, primiparous, or multiparous, with multiparous women predominating in most samples, representing between 40% and 76% of participants (11 , 17 , 18 , 21 , 23 , 26 , 29 , 32). Employment status (employed/unemployed) was reported in at least ten studies (11 , 14 , 15 , 21 - 23 , 25 , 27 , 29 , 32). Marital status, when provided, was categorized as "with partner" or "without partner," with over 80% of women reporting a stable partnership (9 , 10 , 13 , 32). Finally, ethnic or cultural background was reported mainly in multicentre studies or in countries with highly diverse populations (7 - 9 , 11 , 13 , 15 , 21 , 22 , 24 , 27 - 29 , 32). Variables related to oral health knowledge. In studies that specified the scoring system, knowledge scores ranged between 0.13 and 8.39 out of a maximum of 12 points, or between 40% and 82.8% correct responses, depending on the format and scoring criteria used in each questionnaire. The questionnaires covered topics such as oral hygiene during pregnancy, mother-to-child bacterial transmission, prevention of early childhood caries, dental visits, nutrition, and fluoride use. In most studies, approximately two-thirds, knowledge levels were rated as low or moderate, whereas only a few investigations conducted in Saudi Arabia (18 , 22), Malaysia (29), Poland (17), and Italy (15) reported high levels of adequate knowledge, exceeding 70% correct responses. Type and structure of questionnaires. The number of items varied between 14 (30) y 42 (27), with a predominance of closed-ended items with multiple-choice responses. Non-validated instruments were used mainly in studies conducted in Asia (10 , 12 , 14 , 16 - 18) and in America (9 , 11 , 28), whereas validated questionnaires originated from more recent European and Asian contexts such as Poland (17), Italy (15), Iran (31), and Malaysia (29). Some studies incorporated thematic subscales distinguishing maternal oral health knowledge from child oral health knowledge, as observed in the studies by Gavic et al. (20) and Hammad et al. (22). 3. Result of the Quality Analysis The results obtained were statistically analysed in all included studies. After applying the Newcastle-Ottawa Scales (NOS), the following findings were observed: Longitudinal studies. Two studies did not use randomized samples (16 , 24), and one did not provide evidence that the outcome or condition was absent in participants at baseline (16). Cross-sectional studies. The studies were classified as follows: 2 displayed very good quality (27 , 32); 4, good quality (7 , 13 , 17 , 18); 12, satisfactory quality (9 , 10 , 14 , 15 , 19 , 20 , 22 , 25 , 26 , 29 - 31); and 5, unsatisfactory quality (11 , 12 , 21 , 23 , 28) . Most of the included studies had limitations in the areas of 'non-respondents', 'comparability' and 'validated measurement tools'. A lack of information regarding response rates and the characteristics of non-respondents implies a risk of selection bias, as it prevents an adequate assessment of whether the respondents were representative of the target population. Cross-sectional studies (n = 23) demonstrated weaknesses in this area; 11 of these studies used non-validated, self-developed questionnaires, which could introduce measurement error through ambiguous items and potentially lead to an underestimation of true knowledge deficits. The detailed results of this assessment are presented in Table 3 and Table 4.

## Discussion

1. Main findings The results of this review confirm the existence of a significant gap in pregnant women's knowledge, attitudes, and practices regarding oral health. The studies show clear associations between knowledge level and sociodemographic variables such as educational attainment (11 , 13 , 17 , 18 , 20 , 22 , 24 , 27 , 29 , 31), employment status (22 , 27 , 32), cultural background (13 , 27), and parity (9 , 11 , 27). In this regard, Barbieri et al. (11) found higher knowledge among multiparous women, whereas Llena et al. (27). observed the opposite, and Baker et al. (9) found no significant differences between groups. Hormonal changes during pregnancy, together with dietary modifications, are identified as factors that may exacerbate oral conditions and affect fetal health (36). However, the degree of awareness among pregnant women regarding this relationship varies. Gaffar et al. (18), Hans et al. (23), and George et al. (21) reported that fewer than 50% of women recognize the link between oral health and general or fetal well-being, whereas Chawla et al. (24) and Gaszyska et al. (19), found higher levels of awareness, likely due to differences in education, access to information, or methodological approaches. Most pregnant women are unaware of the association between periodontitis and gestational complications, such as preeclampsia, low birth weight, or preterm delivery, despite periodontitis affecting up to 40% of pregnant women (36 , 37). Ten of the included studies addressed this topic, and none reported more than 50% correct responses (7 , 10 , 14 , 15 , 20 - 23 , 27 , 28). This lack of awareness is consistent with uncertainty among healthcare professionals, including gynecologists and obstetricians, which Montoya et al. (38) attribute to the complexity and lack of consensus regarding the causal relationship between periodontal disease and adverse pregnancy outcomes. 2. Comparison with previous literature Regarding dental caries, the studies consistently indicate insufficient knowledge about its etiology and transmissible nature. Adeniyi et al. (8) and Barbieri et al. (11) found that many participants were unfamiliar with the causes of caries, whereas Gaffar et al. (18), Chawlowska et al. (25), and George et al. (21) observed that most women were unaware of the transmission of cariogenic bacteria from mother to child, such as Streptococcus mutans (3). This lack of knowledge may lead to negligent attitudes toward preventive care for both mother and child, compounded by the underestimation of the importance of the primary dentition (19 , 26) 3. Implications for clinical practice and public health Although international clinical guidelines highlight the need to integrate oral health education into prenatal care (39), the studies agree that pregnant women seldom seek dental advice or receive treatment during pregnancy. In the United States, only 44.7% attend dental visits (40), and in Australia the figure drops to 30% (21). Frequent barriers include the cost of visits (17 , 21 , 28 , 30), low perceived risk or importance (21 , 31), the belief that they already have good oral health (9 , 10 , 19 , 21), and fear regarding the supposed harmfulness of dental treatment during pregnancy (21 - 23 , 28 , 30 , 31). Persistent myths include the belief that "a tooth is lost with each pregnancy" (13), "the fetus extracts calcium from the mother's teeth" (16), for that "caries are inevitable during pregnancy" (14). With respect to hygiene habits, although toothbrushing is widely practiced, the use of dental floss (18 , 21 , 29 , 30), mouthrinses (21 , 29), and fluoride (17 , 27 , 29) remains limited. This gap between knowledge and practice underscores the need to strengthen health education, as maternal habits directly influence children's oral health. In line with WHO, ADA, and AAPD recommendations, establishing a "dental home" before the child's first year of life and initiating toothbrushing with fluoride toothpaste upon eruption of the first tooth are advised (4). However, pregnant women's knowledge of these recommendations varies widely: Barbieri et al. (11) and Hammad et al. (22) reported that more than 60% of women knew when to begin brushing, while Bhaskar et al. (12) and Cagetti et al. (15) found rates below 40%. Regarding fluoride, Llena et al. (27) reported that only 28% were aware of its preventive effect, and Lakshmi et al. (26) and Gaszyska et al. (19) found that over 80% lacked this knowledge. The timing of the first dental visit is another area of deficiency: in the studies by Chawlowska et al. (17), George et al. (21), Hammad et al. (22), and Llena et al. (27), fewer than 33% responded correctly. Finally, sources of information about oral health vary across contexts. Gaszyska et al. (19) identified physicians and dentists as the main references (58%), followed by magazines and media (57%) and the internet (30%). In contrast, Gaffar et al. (18) and Chawlowska et al. (17) found that information mainly came from the internet and family members, with limited involvement of healthcare professionals. This educational gap reflects missed opportunities in prenatal care, associated with lack of time, limited training, or uncertainty regarding responsibility among gynecologists and midwives (41 - 47). 4. Limitations of included studies Among the limitations of this review, language restrictions (English, French, Italian, Spanish) may have introduced geographical publication bias by favouring studies from countries with stronger English-language research infrastructure, potentially underrepresenting non-Western contexts that publish primarily in local languages (e.g., additional sub-Saharan African or South Asian studies in Portuguese, Arabic, or regional dialects). Exclusion of non-indexed sources and grey literature (theses, institutional reports, conference proceedings) risks missing unpublished or locally disseminated evidence from low-resource settings, where oral health knowledge gaps may be more pronounced due to limited research dissemination capacity. Moreover, estimating overall levels of knowledge, attitudes, or practices proved challenging due to population and sociocultural heterogeneity, as well as methodological variability among included studies. These factors may contribute to underrepresentation bias toward contexts with more established research ecosystems. Additionally, methodological weaknesses within individual studies may systematically bias reported knowledge levels. Eleven studies (42%) utilized non-validated, self-developed questionnaires (7 - 12 , 19 , 21 - 23 , 28), potentially introducing measurement error through ambiguous items that overestimate knowledge by failing to capture nuanced gaps. Convenience sampling generated selection bias toward more health-literate participants, artificially elevating scores compared to population-representative samples. Self-reported knowledge, universal across all studies, is susceptible to social desirability bias. These factors contribute to the observed heterogeneity in scores (40-82.8% correct). Higher-quality studies employing validated instruments consistently reported lower knowledge levels, suggesting that methodological weaknesses may underestimate true deficits. Despite these limitations, the convergent pattern of insufficient knowledge across quality strata and regions supports the robustness of the primary findings. Despite these limitations, the findings of this review show that most pregnant women have insufficient knowledge about oral health during pregnancy and early childhood. Myths such as "losing a tooth per pregnancy" or the belief that dental treatments are harmful during gestation persist, and fewer than half of pregnant women are aware of the association between periodontal disease and pregnancy complications. Likewise, knowledge of mother-to-child transmission of cariogenic bacteria and of the appropriate timing to initiate infant oral hygiene remains low. Information available to pregnant women often comes from non-professional sources, such as the internet or magazines, highlighting the need to strengthen health education during pregnancy and to integrate dentists into prenatal care programs, along with specific training of healthcare personnel in perinatal oral health. Although integration of oral health into prenatal care has been proposed, more specific interventions are warranted, such as educational programs led by midwives and gynecologists, targeted informational resources for pregnant women, structured dentist referral pathways, and community-based initiatives aimed at improving access oral health information and care during pregnancy. Additionally validated standardized questionnaires would enable periodic assessment of pregnant women's oral health knowledge, support systematic identification of knowledge gaps, inform the adaptation of preventive programs, and facilitate comparisons across settings and countries.

## Conclusions

In conclusion, the available evidence indicates that pregnant women generally exhibit low to moderate levels of oral health knowledge. Most studies included in this systematic review report limited awareness of pregnancy-related oral changes, the association between periodontal disease and adverse pregnancy outcomes, and the prevention of early childhood caries. Although modest improvements are observed in contexts with active preventive programs or higher educational attainment, substantial knowledge gaps and persistent misconceptions remain, particularly regarding the safety of dental treatments during pregnancy and the initiation of oral care in infancy. These findings underscore the need to strengthen oral health education during pregnancy through greater involvement of healthcare professionals and the integration of dental care into maternal health programs.

## Figures and Tables

**Table 1:** Search Strategy in the Databases.

Databases	Search Strategy
Scopus	(TITLE-ABS-KEY (mother* OR wom* OR mater*) AND TITLE-ABS-KEY (pregnan*) AND TITLE-ABS-KEY (knowledge OR awareness) AND TITLE-ABS-KEY (“oral health”))
WOS	((TI=((mother* OR wom* OR mater*) AND pregnan* AND (knowledge OR awareness)AND “oral health”)) OR TS=((mother* OR wom* OR mater*) AND pregnan* AND (knowledge OR awareness)AND “oral health”)) OR AB=((mother* OR wom* OR mater*) AND pregnan* AND (knowledge OR awareness)AND “oral health”)
Embase	(mother*: ti,ab,kw OR wom*: ti,ab,kw OR mater*: ti,ab,kw) AND pregnan*: ti,ab,kw AND (knowledge:ti,ab,kw OR awareness:ti,ab,kw) AND ‘oral health’: ti,ab,kw
PubMed	(((Mother*[Title/Abstract] OR wom*[Title/Abstract] OR mater*[Title/Abstract]) AND (pregnan*[Title/Abstract])) AND (Knowledge [Title/Abstract] OR awareness [Title/Abstract])) AND (“oral health”[Title/Abstract])

1

**Table 2:** Included studies and general characteristics.

Author/year	Location	Type of study	Participants details	Type of questionnaire (n=nº items)	Level of knowledge
Abiola et al 2011[7]	Nigeria	Cross-sectional	Participants n=453Age 31.32 ± 4.318 yearRace/ ethnicityHausa 1.6%Ibo 21.6%Yoruba 62.5%EducationPrimary 1.8%Secondary 10.8%Polytechnic 33.3%University 53.6%ParityPrimigravidae 53.6%	Not validated	Mean knowledge scores(out of 6):Age category (3.00)Level of education (3.01)Ethnic group (3.01)Trimester (3.04)
Adeniyi et al 2018[8]	Lagos	Longitudinal	Participants n=215Age 29.8±4.8 yearRace/ ethnicityYoruba 55.4%Igbo 23.7%Hausa 2.8%Others 18.1%EducationPrimary/ less 73.5%Secondary 15.3%University 11.2%	Not validatedn=25	Moderate 4.58 (±1.37) out of 7
Baker et al 2016[9]	EE. UU	Cross-sectional	Participants n=454Race/ ethnicity Caucasian 41%Latino 32%Afroamerican 20%EducationPrimary 74% Secondary 39%Marital statusSingle 14%Married 86%	Not validatedn=39	Mean 0.64 SD 0.24 out of 1(64% participants with correct answers)
Balan et al 2018[10]	China	Cross-sectional	Participants n=82Age 31.8±4.5 yearMarital status Married 98.8%EducactionSecondary 46.3%University 41.4%Postgraduate 12.2%	Not validated	27.5 ± 3.2 out of 37 points
Barbieri et al 2018[11]	Brazil	Cross-sectional	Participants n=195Age<30 year 72.3%≥30 year 10.2% Race/ ethnicityCaucasian 26.4%Afroamerican 70.5%Asian 3.1%Education Primary/ secondary 80%Superior 20%Employment statusUnpaid 49.7% Paid 50.3%ParityPrimigravidae 46.2%Multigravidae 53.8%	Not validatedn=20	Low <37% -25.6%Moderate 37-55% -37.5%High 55% - 36.9%
Bhaskar et al 2020[12]	India	Cross-sectional	Participants n=400Age 27 yearsParityPrimigravidae 57.2%Multigravidae 42.8%	Not validatedn=25	Low knowledge 75.5% of pregnant women
Bogges et al 2011[13]	EE. UU	Cross-sectional	Participants n=599Age 29.9 ±6 yearsRace/ ethnicityCaucasian n=253Afroamerican n=126Hispanic n=194EducationPrimary 25.9% Secondary 15.2%University 59%Marital statusSingle 14.1%Married 85.9%	Validatedn=39	6.11 ±1.36 out of 8
Boriboonhirunsarn et al 2023[14]	Thailand	Cross-sectional	Participants n=304Age 30.7 yearsEducactionPrimary 50.3%Secondary/ Technician /University 49.7%Employment statusPaid 53.6%ParityNulliparous 37.5%	Validatedn=35	7.5 out of 15Limited knowledge
Cagetti et al 2024[15]	Italy	Cross-sectional	Participants n=1340Age 31-35 yearsRace/ ethnicityItaly 96%Others 4%Employment statusUnpaid 4.33 %Paid 76.5%Freelance 19.17%	Validatedn=27	Average oral health knowledge and attitudes of children 8.39±1.85 out of 12
Chawla et al 2017[16]	India	Longitudinal	Participants n=112Age 26.71 years	Validated	Mean 0.13 SD1413% correct answers
Chawłowska et al. 2022 [17]	Poland	Cross-sectional	Participants n=400Age 29.5 ±5.3 yearsEducation Primary 4%Secondary 40.3%Technician/ University 55.8%ParityPrimigravidae 33.3%Multigravidae 67.9%	Validated n=30	The total OHK16 score was, on average, 11.4 points(±2.6) (out of 16), denoting mean correctness of 71.4%. -
Gaffar et al 2016[18]	Saudi Arabia	Cross-sectional	Participants n=197Age≤30 years 47.4%≥ 31 years 42.2%Education Secondary 69.6%Technician /University/ Postgraduated 30.4%ParityPrimigravidae 40.9%Multigravidae 59.1%	Validatedn=20	>70% revealed goodoral health knowledge
Gaszyńska et al. 2015	Poland	Cross-sectional	Participants n=1380Age≤24 years 31.9%≥25 years 68.1%	Not validated	About 40% of pregnant women do not have the basic dental knowledge
Gavic et al 2022[20]	Croatia	Cross-sectional	Participantes n=325Age 28.86 ± 4.78 yearsParidadPrimigravidae 57.85%Multigravidae 42.15	Validated	Average oral health during pregnancy: 3.4 out of 7Average oral health of baby: 5 out of 9
George et al 2013 [21]	Australia	Cross-sectional	Participants n=241Ag e28.1 ± 5.6 yearsRace/ ethnicityAustralian 74.3%Foreing 25.7%EducationPrimary/ less 46.1% Technician 30.7% University 22%Employment statusPaid 49.9%ParityPrimigravidae 28.2%Multigravidae 71%	Not validated n=29	79.1% of total correct answers
Hammad et al 2018 [22]	Saudi Arabia	Cross-sectional	Participants n=360Age 30.08 yearsRace/ ethnicitySaudi 88.3%Not Saudi 11.7%Education None 0.8% School 35.8%University 56.4%Postgraduate 7%Employment statusUnpaid 71.4%Paid 28.6%	Not validatedn=21	79.7% good oral health in children8.8% good oral health during pregnancy8.1% good overall
Hans et al 2019[23]	India	Cross-sectional	Participants n=225Age 27.07 ± 3.91 yearsEducaction Primary 27.5%Middle 24.4%High school 19.5%Graduate/Postgraduate 28.4%Employment statusUnpaid 80%Paid 19.9% ParityPrimigravidae 23.11%Multigravidae 76.88%	Not validatedn=19	Number of participants giving the correct responses was significantly less than the incorrect responses (P < 0.001)
Hom et al 2012 [24]	EE. UU	Longitudinal	Participants n=119Age<24 years 77%>24 years 23%Race/ ethnicityCaucasian 44%Afroamerican 39%Native Americans 17%EducationDid not finish high school 25%Secondary 30%University 45%Marital statusSingle 86% Married 13%	Validatedn=15	Mean 4.8 out of 6
Jojo et al 2024[25]	India	Cross-sectional	Participants n=256Age 27.93 4.72 yearsEducationUniversity/ Technician 35.2%Employment statusUnpaid 56.6%	Reliability Karl Pearson (0.87)n=16	66.4% poor knowledge 30.9% average knowledge2.7% had good knowledge
Lakshmi et al 2020[26]	India	Cross-sectional	Participants n=606Age 2.8 ± 3.09 yearsEducationPrimary/ less 53.3%Secondary 38%University/ Technician 8.7%Parity Primigravidae 36.6%Multigravidae 62.4 %	Validatedn=20	55.8% of them had inadequate knowledge (low <7)
Llena et al 2019[27]	Spain	Cross-sectional	Participants n=139Age 31.42 ± 5.43 yearsRace/ ethnicitySpanish 80.6%Foreing 19.4%EducationPrimary 15.8%Secondary 57.6%University/ Technician 16.6%ParityPrimigravidae 56.8%Multigravidae 43.2%Employment statusUnpaid 43.2%Paid 56.8%	Validatedn=42	Low 44.6%Moderate 55.4%
Naavaal et al 2022[28]	EE. UU	Cross-sectional	Participants n=187Age 31.6 ±5.89 yearsRace/ ethnicityAfroamerican 79%Hispanic 6%Not Hispanic 94%Education Primary/ less 60%Secondary/ University 40%	Not validated	Women with private insurance 3.6 out of 5 (SD 0.71)Women with Medicaid 2.9 (SD 1.11)Women without insurance 3.2 (SD 1.21)
Niazi et al 2023[29]	Malaysia	Cross-sectional	Participants n=203Age≤35 years 77.3%>35 years 22.7%Race/ ethnicityMalay 99%Others 1%EducationPrimary 3%Secondary 27.6%University/ Postgraduate 69.5%Employment statusUnpaid 38.4%Paid 61.6%ParityNulliparous 30.5%Primigravidae 27.1%Multigravidae 42.4%	Validated	Level of knowledgeAdequate (54.2%) mean 82.8 (± 5.46) out of 96Moderate (31%) mean 68.0 (± 4.27) out of 73.1Inadequate (14.8%) mean 51.4 (±6.79) out of 59.2
Sajjan et al 2015[30]	India	Cross-sectional	Participants n=332Age≤24 years 50%≥25 years 50%	Validatedn=14	1.36±1.12 out of 5
Seyyedi et al 2023[31]	Iran	Cross-sectional	Participants n= 96Age 29.11 ± 6.80 yearsEducationNone 8Primary 56Secondary 21University/ Postgraduate 11	Validatedn=25	Not favorable, average knowledge score of 2.31 ± 2.01 out of 10
Wassihun et al 2021[32]	Ethiopia	Cross-sectional	Participants n=384Age 27.6 ± 5.16 yearsRace/ ethnicityAri 42.4%Amhara 33.3%Bena 16.1%Mursi 7.6%Others 0.5%EducationPrimary/ less83.9%Secundary 16.1%Marital status Single 4.5%Married 95.6%Employment statusUnpaid 56%Paid 44%Parity Primigravidae 25.5%Multigravidae 74.5%	Validated	Good knowledge 34.1%Poor knowledge 65.9%

2

**Table 3:** Otawa Quality Assessment Scale (NOS).

Author/year	Selection				Comparability	Outcome			Score
Adeniyi et al. 2018 [8]	*	*	*	*	**	*	*	*	8
Chawla et al. 2017 [16]	-	*	*	-	**	*	*	*	7
Hom et al. 2012 [24]	-	*	*	*	**	*	*	*	8

3

**Table 4:** Otawa Quality Assessment Scale (NOS) modified for cross-sectional studies.

Author/year	Selection				Comparability	Outcome		Score
Abiola et al. 2011 [7]	*	*	-	*	**	*	*	7
Baker et al. 2016 [9]	*	-	*	*	*	*	*	6
Balan et al. 2018 [10]	*	*	-	*	-	*	*	5
Barbieri et al. 2018 [11]	-	-	-	*	*	*	*	4
Bhaskar et al. 2020 [12]	*	-	-	*	-	*	*	4
Bogges et al. 2011 [13]	*	-	*	**	*	*	*	7
Boriboonhirunsarn et al. 2023 [14]	*	*	-	**	-	*	*	6
Cagetti et al. 2024 [15]	*	*	-	**	-	*	*	6
Chawłowska et al. 2022 [17]	*	*	-	**	*	*	*	7
Gaffar et al. 2016 [18]	*	*	-	**	*	*	*	7
Gaszyńska et al. 2015 [19]	*	-	*	*	*	*	*	6
Gavic et al. 2022 [20]	*	*	-	**	-	*	*	6
George et al. 2013 [21]	*	-	-	*	-	*	*	4
Hammad et al. 2018 [22]	*	*	-	*	-	*	*	5
Hans et al. 2019 [23]	*	-	-	*	-	*	*	4
Jojo et al. 2024 [25]	*	*	-	*	-	*	*	5
Lakshmi et al. 2020 [26]	*	-	-	**	-	*	*	5
Llena et al. 2019 [27]	*	*	*	**	**	*	*	9
Naavaal et al. 2022 [28]	-	-	-	*	*	*	*	4
Niazi et al. 2023 [29]	-	*	-	**	*	*	*	6
Sajjan et al. 2015 [30]	*	*	-	**	-	*	*	6
Seyyedi et al. 2023 [31]	*	-	-	**	-	*	*	5
Wassihun et al. 2021 [32]	*	*	*	**	**	**	*	10

4

## Data Availability

All data generated or analyzed during this study are included in this published article.
